# CD82-associated exhausted CD8^+^ T cells define prognosis and immunotherapy resistance in colon cancer

**DOI:** 10.3389/fimmu.2025.1731154

**Published:** 2026-01-13

**Authors:** Jingfeng Zhang, Hengwei Cui, Shaoxian Wu, Hongwei Shi, Haitao Wang, Jingting Jiang

**Affiliations:** 1 Department of Gastrointestinal Surgery, The Third Affiliated Hospital of Soochow University, Changzhou, Jiangsu, China; 2 Trauma Center, The Third Affiliated Hospital of Soochow University, Changzhou, Jiangsu, China; 3 Department of Tumor Biological Treatment, The Third Affiliated Hospital of Soochow University, Changzhou, Jiangsu, China; 4 Jiangsu Engineering Research Center for Tumor Immunotherapy, The Third Affiliated Hospital of Soochow University, Changzhou, Jiangsu, China; 5 Institute of Cell Therapy, The Third Affiliated Hospital of Soochow University, Changzhou, Jiangsu, China

**Keywords:** CD82, colon cancer, exhausted CD8^+^ T cells, multi-color immunohistochemistry, prognosis

## Abstract

The regulation of T cell exhaustion within the tumor microenvironment plays a pivotal role in shaping the immune response to cancer and determining the efficacy of immunotherapy. However, the molecular factors governing this process in colon cancer remain poorly understood. This study investigates the expression characteristics and functional significance of the transmembrane protein CD82 in the colon cancer immune microenvironment, with emphasis on its regulatory role in CD8^+^ T cell exhaustion and clinical outcomes. Publicly available transcriptomic datasets were integrated with multiplex immunohistochemistry on colon cancer tissue microarrays to characterize the cell–type–specific distribution of CD82 and its associations with key markers of T cell dysfunction. CD82 expression was markedly increased in tumor-infiltrating immune and epithelial cells compared with normal tissues, particularly within exhausted CD8^+^ T cells. Elevated CD82 levels showed strong positive correlations with canonical exhaustion markers such as programmed cell death protein 1 and T cell immunoglobulin and mucin domain-containing protein 3. Multiplex immunohistochemical analysis further revealed that enrichment of CD82-positive epithelial regions and expansion of the CD82^+^TIM-3^+^PD-1^+^CD8^+^ T cell subset were associated with poor prognosis and were confirmed by multivariate Cox regression as independent risk factors for unfavorable survival. In patients who failed to achieve a complete pathological response following immunotherapy, exhausted CD8^+^ T cells exhibited significantly higher CD82 expression. Single-cell regulatory network analysis identified BATF and BHLHE40 as potential transcriptional regulators of CD82. Collectively, these findings demonstrate that CD82 promotes CD8^+^ T cell exhaustion, contributing to tumor progression and immunotherapy resistance in colon cancer. This study provides novel insight into the molecular mechanisms underlying immune dysfunction and offers a potential therapeutic target for reversing immunosuppression and improving immunotherapy efficacy in colon malignancies.

## 1 Introduction

Colorectal cancer (CRC) is the third most common malignant tumor worldwide, and its progression is closely associated with immune evasion within the tumor microenvironment (TME) (1–4). A hallmark of this process is the functional exhaustion of CD8^+^ T cells, which mediates immune suppression and is characterized by persistent overexpression of inhibitory receptors, including PD-1, TIM-3, and LAG-3, along with impaired effector function (5, 6). Although immune checkpoint blockade (ICB) therapy shows marked efficacy in a subset of CRC patients, primary resistance remains widespread, indicating that the regulatory network underlying T cell exhaustion has not yet been fully defined (7–9).

Recent studies demonstrate that the dynamic evolution of T cell exhaustion is closely correlated with TNM stage progression (10). TNFRSF18 has been identified as a novel marker of CD8^+^ T cell exhaustion in CRC, and its expression, together with the dynamic regulation of CXCL13 and ribosomal stem cell characteristics, shapes the immunosuppressive state of the TME (10). Moreover, the strength of T cell receptor (TCR) signaling is observed to be closely associated with exhaustive differentiation, and alterations in CARD11-mediated signaling are shown to significantly influence TCR clonal diversity and antitumor immune responses (11). These findings underscore the multidimensional regulation of T cell exhaustion and provide important insights for the development of novel immunotherapeutic strategies.

CD82 (also known as KAI1), a member of the tetraspanin transmembrane protein family, was initially identified as a metastasis suppressor, and its downregulation is associated with invasive progression in multiple malignancies (12, 13). Recent studies indicate that CD82 plays a pivotal role in immune cells, particularly T cells, by regulating activation and cytokine production (14). Nevertheless, the specific expression patterns, functional associations, and clinical significance of CD82 within the CRC immune microenvironment, especially among CD8^+^ T cell subsets, remain to be clarified. Previous research has primarily examined CD82 expression in tumor epithelial cells, whereas its dynamic expression within immune cell populations of the TME—particularly CD8^+^ T cell subpopulations—has not been systematically characterized (14–16). In addition, the correlation between CD82 and T cell exhaustion markers lacks multi-omics validation, and whether CD82 directly contributes to the regulation of T cell exhaustion programs remains unresolved.

Building on the current research landscape, this study employs a multi-omics integration strategy combined with spatial molecular pathology techniques to systematically elucidate the regulatory role and clinical significance of CD82 in T cell exhaustion and immunotherapy response in colon cancer (17). Single-cell transcriptomic data from colon cancer, mIHC tissue microarray staining, and clinical prognostic information are integrated to characterize the expression patterns of CD82 across distinct functional states of CD8^+^ T cell subsets. Association analyses and multivariate Cox regression models are applied to assess the spatial distribution of CD82-positive T cell subsets and their prognostic relevance. In addition, we aimed to investigate the expression patterns of CD82 and explore its potential transcriptional regulatory mechanisms in treatment-resistant CD8^+^ T cell populations. This was analyzed using single-cell transcriptomic data from immunotherapy recipients.

This study aims to provide novel biological insights into the regulation of the colon cancer immune microenvironment and to offer an experimental basis for the development of targeted CD82-combined immunotherapeutic strategies.

## 2 Materials and methods

### 2.1 Patients and tissue specimens

The colon adenocarcinoma tissue microarray (catalog no. HColA180Su21) is obtained from Shanghai OUTDO BIOTECH Co., Ltd. (Shanghai, China). It contains 94 colon adenocarcinoma tissue samples and 86 normal colon tissue samples. None of the patients received preoperative neoadjuvant radiotherapy, chemotherapy, or other antitumor adjuvant therapies. All cases are pathologically confirmed as colon adenocarcinoma following surgical resection performed between February 24, 2012, and September 4, 2014. Clinical and pathological data, including sex, age, tumor diameter, TNM stage (AJCC 7th edition), and pathological grade, as well as follow-up information on survival status and duration, are collected for all patients. Follow-up is completed by July 31, 2018. Samples with tissue detachment during processing or with incomplete clinical or follow-up data are excluded.

Follow-up time is defined as the interval from the date of surgery to death or the last follow-up. Only patients with complete follow-up information are included in the survival analysis. The median follow-up duration is calculated and is reported in the Results section.

In total, 86 colon adenocarcinoma tissue samples and 62 normal colon tissue samples are included in the analysis. The study is reviewed and approved by the Clinical Research Ethics Committee at Outdo Biotech (Shanghai, China, SHYJS-CP-1910009).

### 2.2 Single-cell integration, dimensionality reduction, and clustering analysis

Single-cell RNA sequencing datasets GSE205506 and GSE178341 are downloaded from the GEO database. To clarify the clinical context of the GSE205506 dataset, detailed information from the PICC trial (NCT03926338) is incorporated. Patients included in this single-cell RNA-sequencing cohort are enrolled in a randomized phase II study evaluating neoadjuvant PD-1 blockade in locally advanced, dMMR/MSI-H colorectal cancer. All patients receive toripalimab at 3 mg/kg, administered intravenously over 30 minutes on day 1 of each 14-day cycle, for a total of six cycles before curative resection. In the combination arm, celecoxib 200 mg is additionally administered orally twice daily from day 1 to day 14 of each cycle. Surgical resection is planned within four weeks after completion of neoadjuvant therapy. Tissue and blood samples used for single-cell RNA sequencing are obtained from pre-treatment biopsies and post-treatment surgical specimens, while serum samples are collected both before treatment and immediately prior to surgery. In total, 40 samples from 19 patients are analyzed by scRNA-seq, enabling the evaluation of CD82-related T-cell phenotypes within a clearly defined PD-1 blockade treatment context (18).

Raw matrices are imported, and integration objects are constructed using the Read10X and CreateSeuratObject functions in the Seurat package. Highly expressed genes are identified with DUBStepR after log-normalization. CellCycleScoring is applied to calculate cell cycle scores based on the cc.genes dataset, and their influence is regressed during ScaleData. Principal component analysis (PCA) is then performed using highly differentially expressed genes as input features. Batch effects are corrected with Harmony, followed by UMAP-based dimensionality reduction, construction of a nearest-neighbor graph, and unsupervised clustering on selected principal components.

### 2.3 Analysis of CD8^+^ T cell subpopulations

In the integrated cohort, T cells are initially defined by the expression of CD3D, CD3E, and TRAC, alongside low expression of B-cell markers such as MS4A1 and CD79A. CD8^+^ T cell subsets are subsequently selected based on CD8A/B positivity and low CD4 expression. High-mutability genes are identified for these subsets using DUBStepR after log-normalization. Cell cycle scores are calculated by applying CellCycleScoring with the cc.genes dataset, and their influence is regressed in ScaleData. PCA analysis is subsequently performed using highly variable genes as features, with batch effects removed and integrated through the Harmony package. UMAP dimensionality reduction and k-nearest neighbor graph construction are completed on the selected principal components. Unsupervised clustering at a higher resolution is then conducted to identify CD8^+^ T cell subsets. These subsets are annotated based on functional and phenotypic markers, and candidate genes (e.g., CD82) are grouped for visualization analysis.

### 2.4 SCENIC analysis

CD8^+^ T cells are extracted from the integrated dataset, and the Single-Cell Regulatory Network Inference and Clustering (SCENIC) workflow is employed to analyze their transcriptional regulatory networks. First, the cisTarget motif database matching the study species (human hg38, covering transcription start sites ±500 bp and ±10 kb regions) is downloaded as a reference for subsequent regulon enrichment. Low-expression genes are then filtered in the R environment, and the expression matrix along with the transcription factor list is exported using exportsForArboreto. For co-expression network inference, pySCENIC’s GRNBoost2 is used to generate weighted gene-transcription factor networks, which are then imported back into R for further processing. RcisTarget is employed to match co-expression modules with the motif library to identify regulons and their enrichment scores. AUCell is used to convert these scores into Area Under the Curve (AUC) values at the single-cell level to quantify regulon activity. Finally, dimensionality reduction and grouped visualization are performed based on the AUC matrix.

### 2.5 TCGA-COAD data correlation analysis

Pearson correlation analysis is performed using TCGA-COAD RNA-seq data from the GEPIA platform (http://gepia.cancer-pku.cn/) to evaluate the mRNA expression correlations between CD82 and T cell exhaustion-associated markers (CTLA4, FOXP3, TIGIT, CD8A, MRC1 [CD206], PDCD1 [PD-1], HAVCR2 [TIM-3], and LAG3).

### 2.6 mIHC staining

This detection procedure includes steps such as dewaxing, antigen retrieval, application of six antibodies with their corresponding fluorine-labeled cycles (primary antibody incubation, secondary antibody binding, and fluorine labeling), and nuclear counterstaining. The entire staining process is performed using the AlphaXPainter^®^ fully automated staining instrument (Catalog No.: AXT37100041, AlphaXBio, China). The duration and temperature of each reagent incubation step are strictly controlled by the instrument. Dewaxing solution (Catalog No.: DZ2011, Leagene, China) and antigen retrieval solution (Catalog No.: ZLI-9079, ZSGB-BIO, China) expose antigens within the tissue sections. The following antibodies are used: CD82 (clone 1E7A4, 1:1000 dilution, Catalog No.: 66803-1-lg, Proteintech, USA), CD8 (clone 1G2B10, 1:2000 dilution, Cat. No.: 66868-1-Ig, Proteintech, USA), TIM-3 (clone D5D5R™, 1:100 dilution, Catalog No.: 45208S, CST, USA), TCF1 (clone C63D9, 1:200 dilution, Catalog No.: 2203S, CST, USA), PD-1 (clone UMAB199, 1:100 dilution, Cat. No.: ZM0381, ZSGB-BIO, China), and PANCK (clone PDH09-10, 1:2000 dilution, Cat. No.: HA601138, HUABio, China). These antibodies specifically bind to their corresponding proteins in the tissue. The AlphaXTSA^®^ 7-Color Fluorescent Staining Kit (Cat. No.: AXT37100041, AlphaXBio, China) binds to the primary antibody, followed by conjugation of the secondary antibody with fluorescein. After six rounds of labeling, the staining process is completed with DAPI nuclear counterstaining.

### 2.7 Imaging analysis

A whole-slide imaging system with ZEN 3.3 software (ZEISS AXIOSCAN 7, ZEISS, Germany) is used for panoramic multispectral scanning of slides. Images are processed using HALO Pathology Analysis Software (HALO 3.5, IndicaLabs, USA), which employs spectral separation technology to decompose each fluorescent signal into independent channels and archive them as distinct files. A DAPI-stained image is used to generate a binary mask of all viable cells. Expression signals for CD82, CD8, TIM-3, TCF1, and PD-1 are similarly combined with DAPI to construct separate binary masks for cells expressing these target biomarkers. The CK binary mask is then used to count localized tumor cells. PANCK is employed to segment tissue regions, classifying CK-positive contiguous areas identified through histomorphometric multi-imaging as tumor parenchymal zones and CK-negative contiguous areas as stroma zones.

### 2.8 Survival analysis and multifactorial cox regression model

The proportions of each cell subpopulation in the colon cancer tissue microarray are classified into “low expression” and “high expression” groups using X-tile software (version 3.6.1). Cutoff selection is performed under the software’s default settings, including the minimum P-value approach with Monte Carlo–based significance testing (significance level = 0.05), and the optimal cut-point is determined based on the maximal chi-square value. Kaplan-Meier survival curves are then plotted using GraphPad Prism 9.5 software. To further investigate the relationship between these variables and clinical data, univariate and multivariate analyses are performed using Cox proportional hazards regression models, with model fitting achieved through the coxph function.

### 2.9 Statistical analysis

Statistical analysis is performed using GraphPad Prism 9.5 software and RStudio 4.4.2. Chi-square tests are employed to compare the infiltration densities and proportions of various cell subpopulations between normal and tumor tissues, as well as within epithelial and stromal regions of tumor tissues. Log-rank survival analysis was performed to compare overall survival (OS) between postoperative patient groups. Cox regression analysis is applied to determine the prognostic value of each indicator for colon cancer. A *P* value < 0.05 is considered statistically significant.

## 3 Results

### 3.1 CD82 is upregulated in tumor-infiltrating CD8^+^ T cells and correlates with an exhausted phenotype

**Figure 1:**
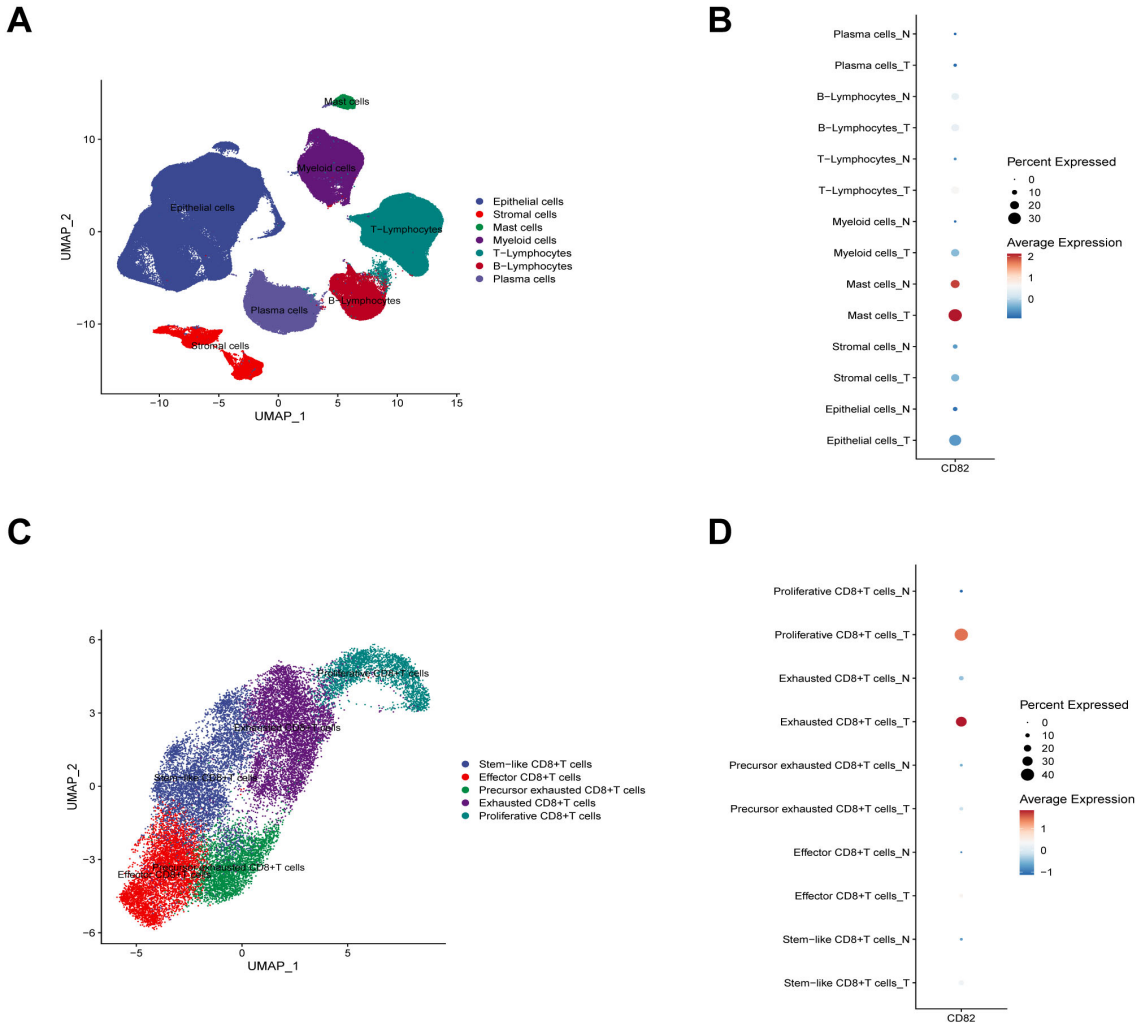
Expression distribution of CD82 in CD8^+^ T cell subsets analyzed by scRNA-seq. **(A)** UMAP visualization of cell types based on scRNA-seq data obtained from the GEO database. **(B)** Dot plot illustrating CD82 expression levels across different cell types in tumor and normal tissues from patients with colon cancer. **(C)** UMAP visualization of CD8^+^ T cell subsets based on scRNA-seq data obtained from the GEO database. **(D)** Dot plot illustrating CD82 expression levels across distinct CD8^+^ T cell subsets in tumor and normal tissues from patients with colon cancer.

Using multi-omics sequencing data from public databases, the expression patterns of CD82 across different cell populations in normal and tumor tissues from human colon cancer patients are systematically analyzed. First, single-cell RNA sequencing (scRNA-seq) data from colon cancer are leveraged to identify seven major cell types in tumor tissues (**Figure 1A**). Comparative analysis reveals that CD82 is expressed in the vast majority of cell types in both tumor and normal tissues. Notably, CD82 expression levels are higher in tumor tissue T cells compared to normal tissue. Additionally, CD82 expression is elevated in myeloid cells, mast cells, stromal cells, and epithelial cells within tumor tissue relative to normal tissue. In contrast, CD82 expression shows no significant difference between tumor and normal tissues in plasma cells and B cells (**Figure 1B**).

Further analysis of CD8^+^ T cell subsets identifies five functionally distinct subpopulations (**Figure 1C**). At the CD8^+^ T cell subset level, CD82 expression shows an increasing trend in exhausted and proliferating CD8^+^ T cells within tumor tissue compared to normal tissue. However, no significant differences in CD82 expression are observed between tumor and normal tissues for the exhausted precursor, effector, or stem cell-like CD8^+^ T cell subsets (**Figure 1D**).

### 3.2 CD82 expression shows a significant positive correlation with T cell exhaustion markers

**Figure 2:**
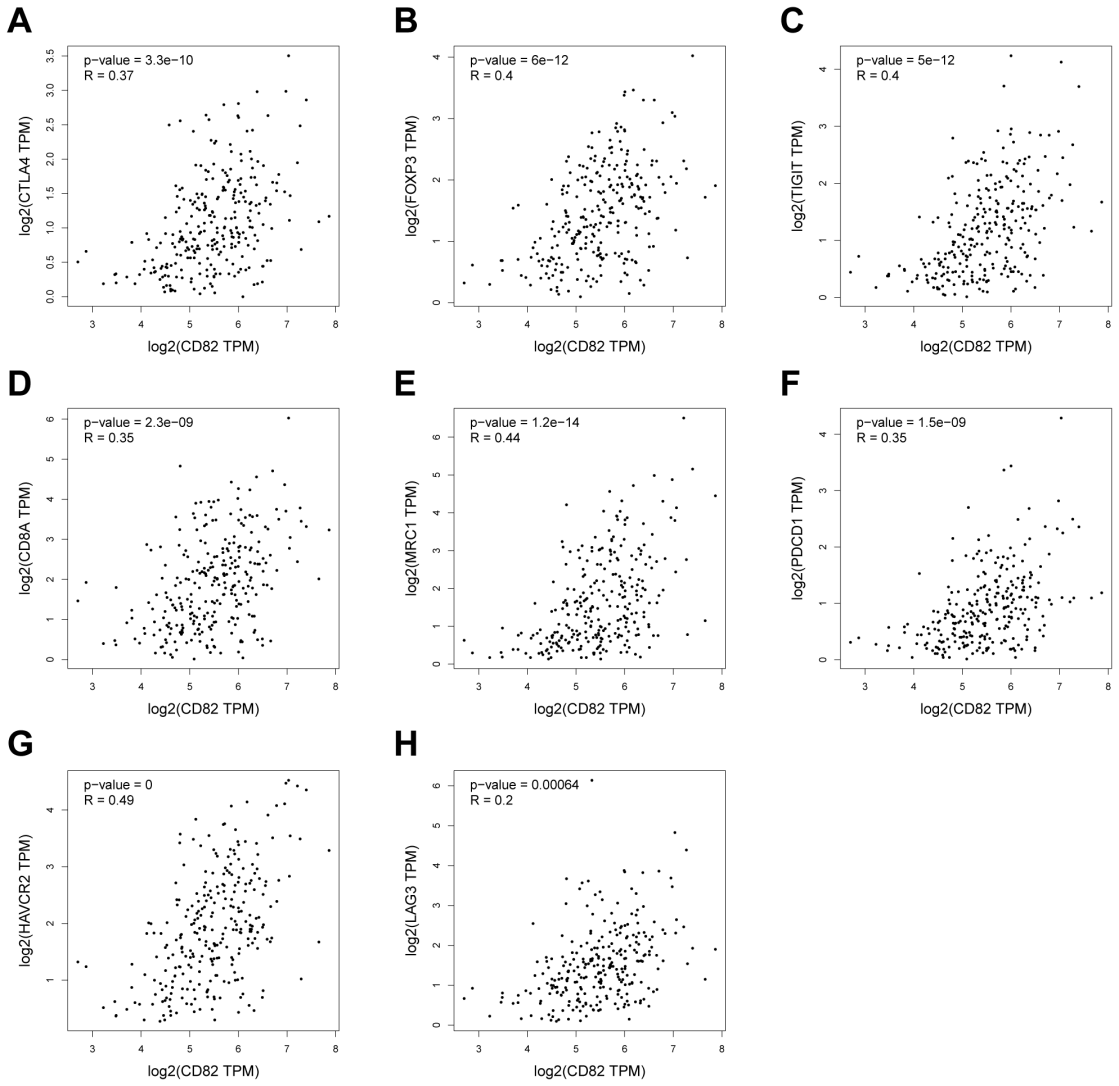
Correlation analysis of CD82 expression with T cell exhaustion markers in TCGA-COAD. **(A–H)** Scatter plots illustrating the correlations between CD82 expression and T cell exhaustion–associated molecules in colon cancer tissues: **(A)** CTLA4, **(B)** FOXP3, **(C)** TIGIT, **(D)** CD8A, **(E)** MRC1 (CD206), **(F)** PDCD1 (PD-1), **(G)** HAVCR2 (TIM-3), and **(H)** LAG3. Correlations are assessed using Pearson’s correlation analysis (all *P* < 0.001).

Using scRNA-seq data from colon adenocarcinoma available in public databases, the correlation between CD82 expression and key T cell exhaustion markers is analyzed. Pearson correlation analysis reveals that CD82 expression correlates with CTLA4 (*r* = 0.37, *P* < 0.001), FOXP3 (*r* = 0.40, *P* < 0.001), TIGIT (*r* = 0.40, *P* < 0.001), CD8A (*r* = 0.35, *P* < 0.001), MRC1 (CD206, *r* = 0.44, *P* < 0.001), PDCD1 (PD-1, *r* = 0.35, *P* < 0.001), HAVCR2 (TIM-3, *r* = 0.49, *P* < 0.001), and LAG-3 (*r* = 0.20, *P* < 0.001) (**Figure 2A–H**). These results suggest that CD82 may participate in the regulatory networks of T cell exhaustion (6, 19–22).

### 3.3 An increased proportion of CD8^+^CD82^+^ T cells correlates with poor prognosis in colon cancer patients

**Figure 3:**
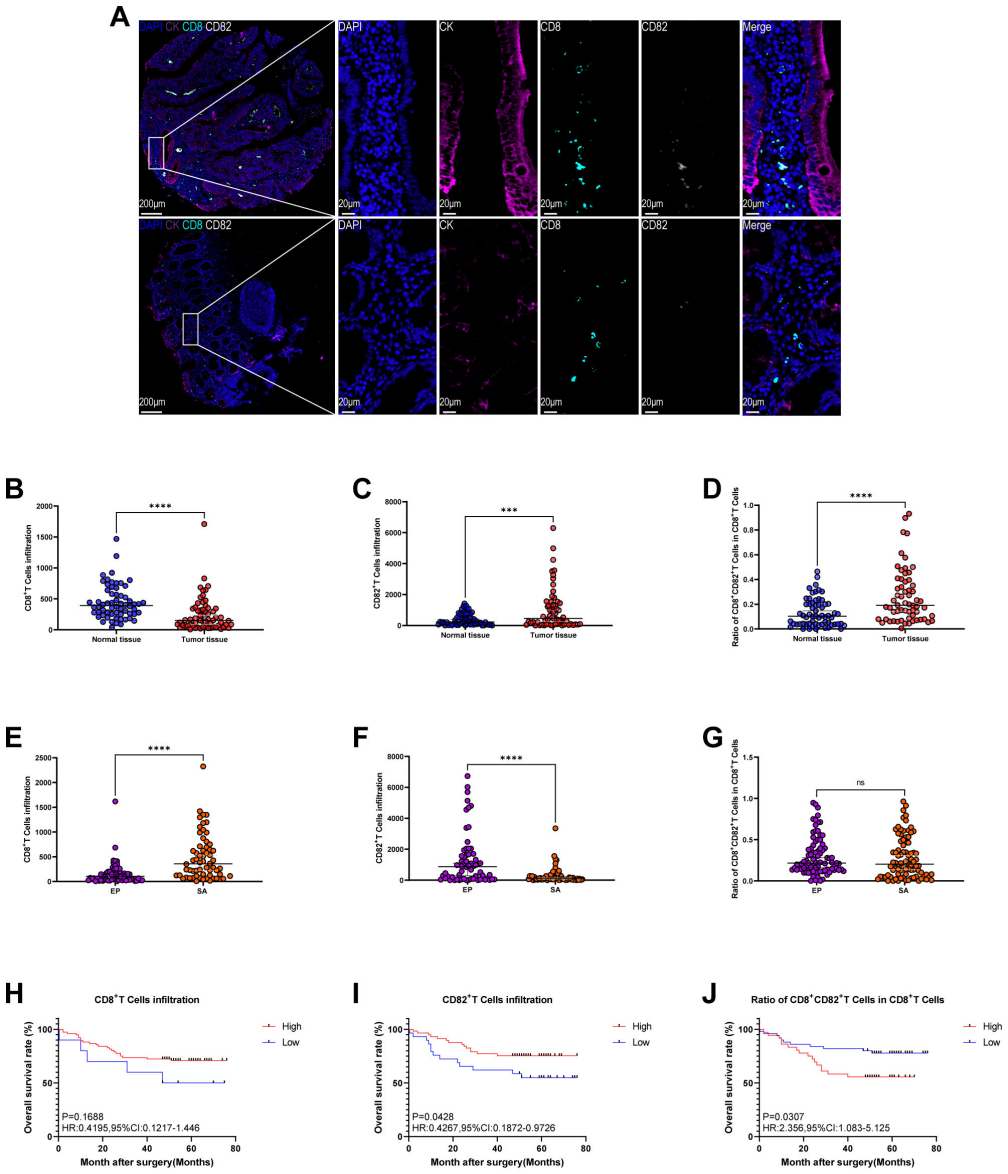
Expression of CD82 in colon cancer tissue microarrays (TMA) and its prognostic significance. **(A)** Representative multiplex immunohistochemical (mIHC) staining and single-channel images of colon cancer TMAs (DAPI, blue; CK, purple; CD8, cyan; CD82, white). **(B–D)** Comparisons between normal and tumor tissues: **(B)** infiltration density of CD8^+^ T cells, **(C)** infiltration density of D82^+^ T cells, and **(D)** proportion of the CD8^+^CD82^+^ subset within total CD8^+^ T cells. **(E–G)** Comparisons between epithelial and stromal regions within tumor tissues: **(E)** infiltration density of CD8^+^ T cells, **(F)** infiltration density of CD82^+^ T cells, and **(G)** proportion of the CD8^+^CD82^+^ subset within total CD8^+^ T cells. **(H–J)** Kaplan–Meier survival curves for colon cancer patients stratified by: **(H)** CD8^+^ T cell infiltration density, **(I)** CD82^+^ T cell infiltration density, and **(J)** proportion of the CD8^+^CD82^+^ subset within total CD8^+^ T cells. Optimal cutoff values for high and low expression groups are determined using X-tile software. *P* values for survival differences are calculated using the Kaplan–Meier method, and HR with 95%CI are estimated by univariate Cox proportional hazards regression analysis. ***P < 0.001; ****P < 0.0001.

Immunohistochemical staining of colon cancer and normal tissues visually demonstrates the spatial distribution of CD8^+^ T cells, CD82^+^ cells, and CK^+^ epithelial cells (**Figure 3A**: DAPI, blue; CK, purple; CD8, cyan; CD82, white). Quantitative analysis reveals that, compared with normal tissue, colon cancer tissue exhibits significantly reduced infiltration of CD8^+^ tumor-infiltrating lymphocytes (TILs) (**Figure 3B**, *P* < 0.0001). In contrast, CD82^+^ TILs infiltration is significantly increased (**Figure 3C**, *P* < 0.001), and the proportion of CD8^+^CD82^+^ T cell subpopulations within CD8^+^ T cells is markedly elevated (**Figure 3D**, *P* < 0.0001).

Further comparison of the distribution differences between these cell subsets in the epithelial and stromal zones within tumor tissues reveals that CD8^+^ T cell density is significantly higher in the stromal zone than in the epithelial zone (**Figure 3E**, *P* < 0.0001), whereas CD82^+^ T cell infiltration is significantly higher in the epithelial zone than in the stromal zone (**Figure 3F**, *P* < 0.0001). The proportion of CD8^+^CD82^+^ T cell subsets within CD8^+^ T cells shows no significant difference between the epithelial and stromal regions (**Figure 3G**). CD8^+^ T cells are significantly enriched in the stromal region, suggesting that they may be excluded from the tumor core. This phenomenon may relate to tumor immune evasion mechanisms, such as physical and biochemical barriers, including abnormal vascular architecture, dense extracellular matrix, or inhibitory signaling molecules that impede T cell infiltration (23). In contrast, the marked aggregation of CD82^+^ cells in the epithelial region suggests that CD82 may play a role in helping T cells adapt to the immunosuppressive microenvironment of the tumor core, possibly associated with their exhausted or dysfunctional state.

Given the role of tumor-infiltrating CD8^+^ T cells in tumor immune surveillance and their prognostic value, the relationship between CD8^+^ T cell subsets and survival in colon cancer patients is evaluated (24). A total of 86 colon adenocarcinoma patients are included in the survival analysis. With follow-up completed on July 31, 2018, the median follow-up duration is 52.0 months (range: 0.37–76.0 months). Kaplan-Meier survival analysis reveals that overall CD8^+^ TILs infiltration is not significantly correlated with OS (**Figure 3H**). In contrast, higher infiltration of CD82^+^ TILs is associated with significantly improved OS (HR = 0.4267, 95% CI: 0.1872–0.9726, *P* = 0.0428; **Figure 3I**). Notably, a lower proportion of CD8^+^CD82^+^ T cells within CD8^+^ T cells is associated with significantly superior OS (HR = 2.356, 95% CI: 1.083–5.125, *P* = 0.0307; **Figure 3J**).

### 3.4 CD8^+^CD82^+^TIM-3^+^PD-1^+^ T-cell infiltration correlates with poor prognosis and serves as an independent risk factor for colon cancer

**Figure 4:**
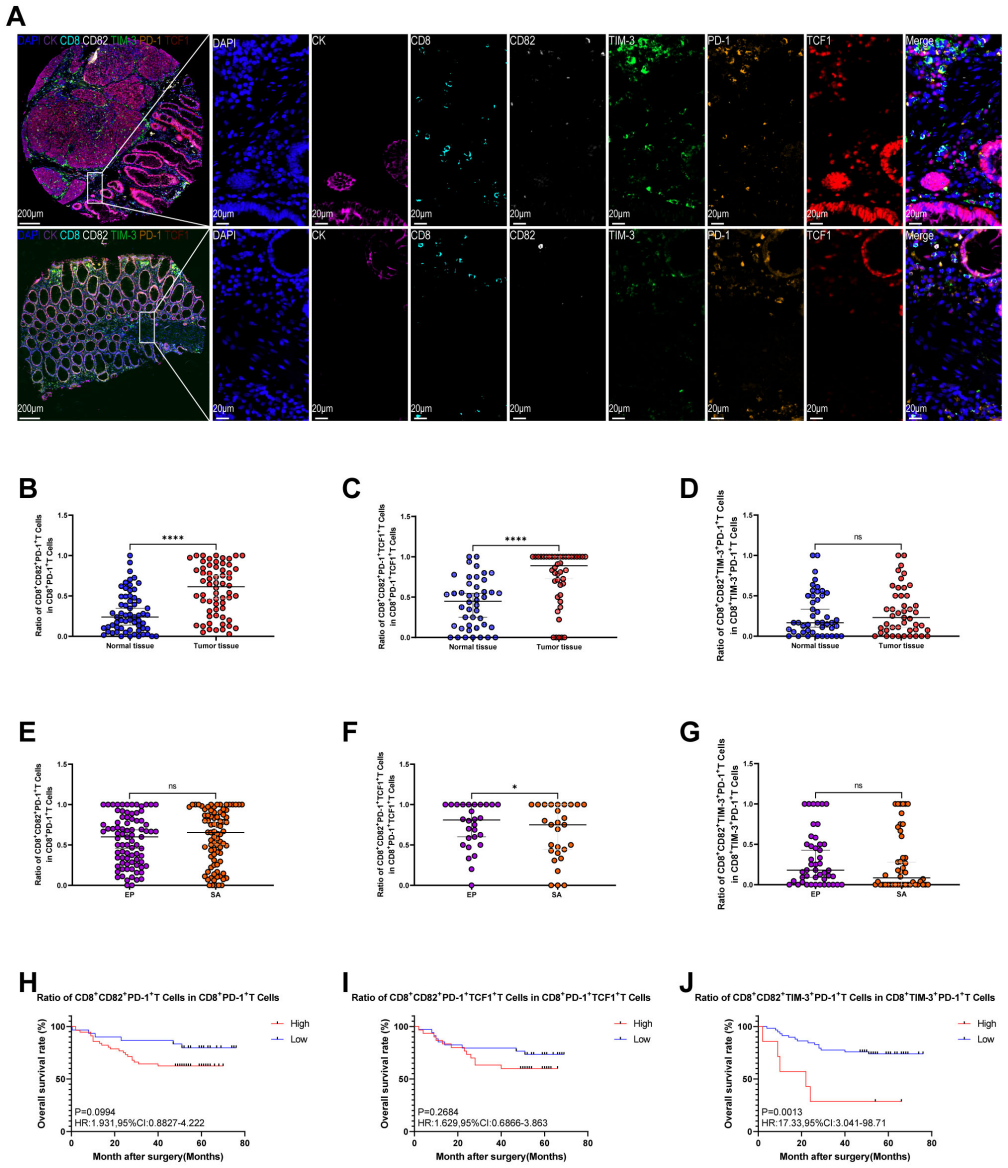
Co-expression of CD82 and exhaustion markers in colon cancer TMA and their prognostic significance. **(A)** Representative mIHC staining and single-channel images (DAPI, blue; CK, purple; CD8, cyan; CD82, white; TIM-3, green; PD-1, orange; TCF1, red). **(B–D)** Comparisons between normal and tumor tissues: **(B)** proportion of CD8^+^CD82^+^PD-1^+^ cells within CD8^+^PD-1^+^ T cells, **(C)** proportion of CD8^+^CD82^+^PD-1^+^TCF1^+^ cells within CD8^+^PD-1^+^TCF1^+^ T cells, and **(D)** proportion of CD8^+^CD82^+^TIM-3^+^PD-1^+^ cells within CD8^+^TIM-3^+^PD-1^+^ T cells. **(E–G**) Comparisons between epithelial and stromal regions within tumor tissues: **(E)** proportion of CD8^+^CD82^+^PD-1^+^ cells within CD8^+^PD-1^+^ T cells, **(F)** proportion of CD8^+^CD82^+^PD-1^+^TCF1^+^ cells within CD8^+^PD-1^+^TCF1^+^ T cells, and **(G)** proportion of CD8^+^CD82^+^TIM-3^+^PD-1^+^ cells within CD8^+^TIM-3^+^PD-1^+^ T cells. **(H–J)** Kaplan–Meier survival curves for colon cancer patients stratified by: **(H)** proportion of CD8^+^CD82^+^PD-1^+^ cells within CD8^+^PD-1^+^ T cells, **(I)** proportion of CD8^+^CD82^+^PD-1^+^TCF1^+^ cells within CD8^+^PD-1^+^TCF1^+^ T cells, and **(J)** proportion of CD8^+^CD82^+^TIM-3^+^PD-1^+^ cells within CD8^+^TIM-3^+^PD-1^+^ T cells. Optimal cutoff values for high and low expression groups are determined using X-tile software. *P* values for survival differences are calculated using the Kaplan–Meier method, and HR with 95%CI are estimated by univariate Cox proportional hazards regression analysis. *P < 0.05; ****P < 0.0001.

mIHC staining detects the expression of CD8, CD82, TIM-3, PD-1, TCF1, and CK in colon cancer and normal tissues (**Figure 4A**). Analysis reveals that, compared with normal tissue, the proportion of CD8^+^CD82^+^PD-1^+^ T cell subsets (**Figure 4B**, *P* < 0.0001) and CD8^+^CD82^+^PD-1^+^TCF1^+^ T cell subsets (**Figure 4C**, *P* < 0.0001) is significantly elevated in colon cancer tissue. However, the proportion of CD8^+^CD82^+^TIM-3^+^PD-1^+^ T cell subpopulations shows no significant difference between tumor and normal tissues (**Figure 4D**).

Spatial distribution analysis (epithelial vs. stromal regions) reveals that the proportion of CD8^+^CD82^+^PD-1^+^TCF1^+^ T cell subpopulations is significantly higher in the epithelial region than in the stromal region (**Figure 4F**, *P* < 0.05). In contrast, the proportion of CD8^+^CD82^+^PD-1^+^ T cell subpopulations (**Figure 4E**) and CD8^+^CD82^+^TIM-3^+^PD-1^+^ T cell subpopulations (**Figure 4G**) shows no significant difference between epithelial and stromal regions.

Survival analysis reveals no significant correlation between the proportion of CD8^+^CD82^+^PD-1^+^ T cell subpopulations (**Figure 4H**) or CD8^+^CD82^+^PD-1^+^TCF1^+^ T cell subpopulations (**Figure 4I**) and OS in patients. In contrast, a lower proportion of CD8^+^CD82^+^TIM-3^+^PD-1^+^ T cell subpopulations is associated with significantly prolonged OS (HR = 17.33, 95% CI: 3.041–98.71, *P* = 0.0013; **Figure 4J**).

Multivariate Cox regression models, adjusted for confounders, confirm that higher pathological grade (III–IV vs. I–II) (*P* < 0.001), TNM stage III–IV (vs. I–II) (*P* < 0.01), and a high CD8^+^CD82^+^TIM-3^+^PD-1^+^ subset proportion (*P* < 0.05) are independent risk factors for OS (**Table 1**). This model provides critical evidence for clinical prognosis assessment and personalized treatment.

**Table 1:** Univariate and multivariate Cox regression analyses of overall survival in patients with colon cancer.

Clinical parameters	Single-factor analysis		Multifactor analysis	
	HR (95% CI)	P value	HR (95% CI)	P value
Gender (male)/(female)	0.748(0.310-1.806)	0.519	1.152(0.413-3.215)	0.787
Age (≧60)/(<60)	0.510(0.212-1.226)	0.133	0.444(0.158-1.251)	0.124
Tumor size (≧5)/(<5)	0.456(0.190-1.098)	0.080	0.374(0.132-1.058)	0.064
Pathological stage ((III+VI)/(I+II))	3.255(1.346-7.874)	**0.009**	5.888(2.168-15.994)	**<0.001**
TNM stage ((III+VI)/(I+II))	5.581(2.135-14.590)	**<0.001**	4.267(1.520-11.977)	**0.006**
Ratio of CD8^+^CD82^+^T Cells in CD8^+^T Cells (high/low)	3.188(1.269-8.011)	**0.014**	0.566(0.121-2.661)	0.471
Ratio of CD8^+^CD82^+^PD-1^+^T Cells in CD8^+^PD-1^+^T Cells (high/low)	2.503(0.836-7.493)	0.101	3.988(0.760-20.921)	0.102
Ratio of CD8^+^CD82^+^TIM-3^+^PD-1^+^T Cells in CD8^+^TIM-3^+^PD-1^+^T Cells (high/low)	4.635(1.666-12.890)	**0.003**	5.810(1.439-23.456)	**0.013**

Bold values indicate statistical significance (P < 0.05).

### 3.5 High expression of CD82 in exhausted CD8^+^ T cell subpopulations correlates with immunotherapy resistance in colon cancer

**Figure 5:**
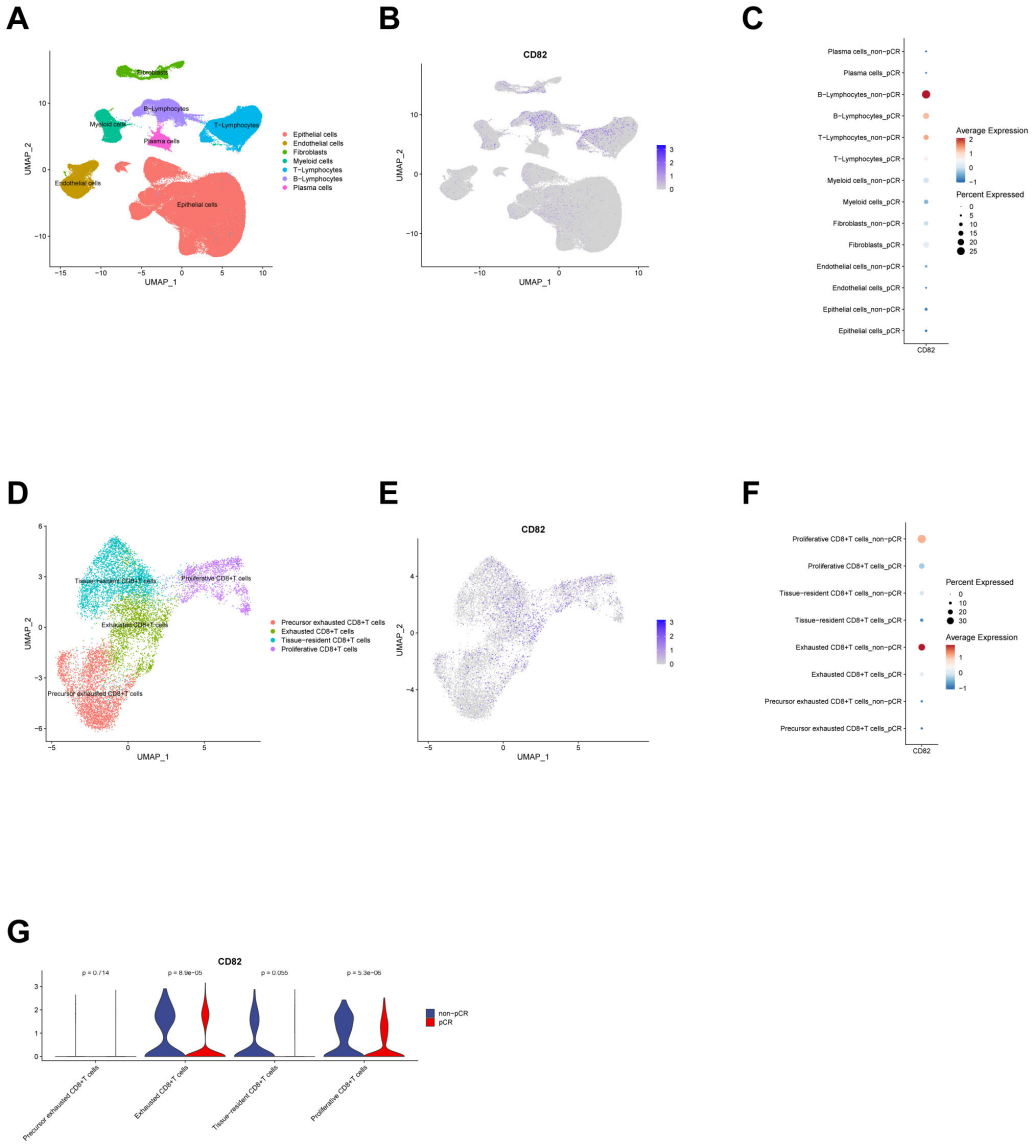
scRNA-seq analysis and expression distribution of CD82 in CD8^+^ T-cell subpopulations following immunotherapy. **(A, B)** UMAP visualization of cell types **(A)** and CD82 expression distribution **(B)** based on scRNA-seq data from GEO datasets of colon cancer patients treated with immunotherapy. **(C)** Dot plot showing differences in CD82 expression levels across cell types between pathological complete response (pCR) and non-pCR groups after immunotherapy. **(D, E)** UMAP visualization of CD8^+^ T-cell subsets **(D)** and corresponding CD82 expression distribution **(E)**. **(F)** Scatter plot comparing CD82 expression levels among distinct CD8^+^ T-cell subsets between pCR and non-pCR groups post-immunotherapy. **(G)** Violin plot illustrating CD82 expression differences among CD8^+^ T-cell subsets between pCR and non-pCR groups following immunotherapy.

Based on scRNA-seq data from colon cancer patients receiving immunotherapy, seven major cell types are first identified in tumor tissues (**Figure 5A**). CD82 is expressed in nearly all cell types, with more pronounced expression observed in T lymphocytes and B lymphocytes (**Figure 5B**). A comparison between the pathological complete response (pCR) and non-pCR groups shows that CD82 expression in T lymphocytes, B lymphocytes, and myeloid cells is higher in the non-pCR group than in the pCR group (**Figure 5C**). In contrast, no significant differences in CD82 expression are observed between the two groups in plasma cells, fibroblasts, endothelial cells, or epithelial cells (**Figure 5C**).

Subcloning of CD8^+^ T cells identifies four distinct subsets (**Figure 5D**), with CD82 expression detected across all subsets (**Figure 5E**). Subpopulation-specific analysis reveals that, in the non-pCR group, CD82 expression levels are higher in exhausted CD8^+^ T cells (**Figures 5F, G**) and proliferating CD8^+^ T cells (**Figures 5F, G**) compared to the pCR group. No significant difference in CD82 expression is observed between the two groups in tissue-resident CD8^+^ T cells and pre-exhausted CD8^+^ T cells (**Figures 5F, G**).

### 3.6 BATF and BHLHE40 are identified as potential transcriptional regulators of CD82 in CD8^+^ T cells and may influence the response to immunotherapy

**Figure 6:**
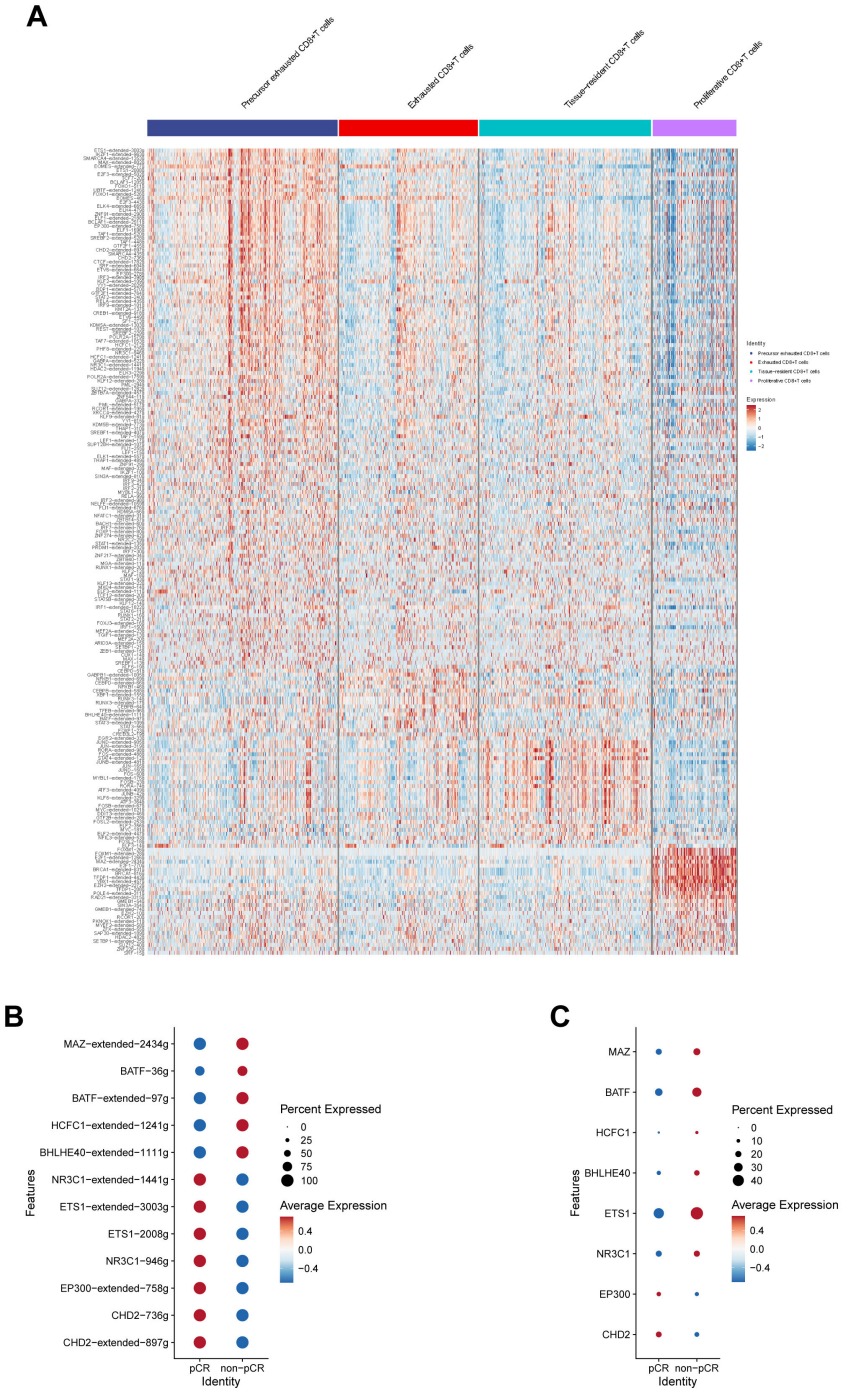
Single-cell regulatory network inference and clustering analysis of CD8^+^ T cells. **(A)** Heatmap showing regulon activity (AUCell AUC values) across CD8^+^ T-cell subsets. **(B)** Differentially expressed transcriptional regulators between pCR and non-pCR groups predicted to bind the promoter or enhancer regions of the CD82 gene. **(C)** Comparison of mRNA expression levels of candidate regulatory factors between pCR and non-pCR groups.

To investigate the relationship between regulator activity and immunotherapy response in CD8^+^ T cells, regulon activity (AUCell AUC values) across CD8^+^ T cell subsets is first visualized using heatmaps (**Figure 6A**). Further screening identifies candidate regulators that are differentially expressed between the pCR and non-pCR groups, with predicted binding sites located within the promoter or enhancer regions of the CD82 gene (**Figure 6B**). Differential analysis of these candidates shows a high consistency between the regulatory activity of BATF and BHLHE40 (**Figure 6B**) and their expression levels (**Figure 6C**). In conclusion, this study suggests that BATF and BHLHE40 act as transcriptional regulators of CD82, thereby influencing the immunotherapy response by modulating CD82 expression.

## 4 Discussion

This study systematically analyzes the expression characteristics of CD82 in the colon cancer immune microenvironment and its clinical significance, revealing a previously underappreciated role of this molecule in regulating T cell function (14). Compared to normal tissues, CD82 expression is significantly upregulated in tumor-infiltrating T cells, particularly CD8^+^ T cells, and shows strong positive correlations with T cell exhaustion markers such as PD-1, TIM-3, and CTLA-4. These findings position CD82 as a key molecule associated with T cell dysfunction within the tumor immune microenvironment, thereby expanding the current understanding of the complexity of the immune checkpoint network (25). Notably, while previous studies have primarily focused on the metastasis-inhibitory function of CD82 in epithelial tumor cells, this research is the first to elucidate, at single-cell resolution and in spatial dimensions, the specific overexpression and clinical significance of CD82 in tumor-infiltrating lymphocytes, particularly within exhausted CD8^+^ T cell subsets (26–31). These results not only provide a novel perspective on the immune functions of CD82 but also enhance our understanding of the immune escape mechanisms in colon cancer.

We find that increased infiltration of CD82^+^ TILs in tumor tissues is significantly associated with improved overall survival in patients, whereas a higher proportion of CD8^+^CD82^+^ double-positive T cells is linked to poor prognosis. This seemingly contradictory phenomenon may reflect the functional diversity of CD82 across different cell types and microenvironmental contexts. As a known tumor metastasis inhibitor, CD82 exerts positive effects in cancer cells by suppressing migration and invasion. Conversely, its expression increases significantly in T cells, particularly within exhausted subsets, suggesting that CD82 may contribute to maintaining or exacerbating T cell dysfunction (28). Multiplex immunohistochemical analysis further reveals the enrichment of CD82^+^ T cells in the epithelial zone, an area typically characterized by stronger immunosuppressive signaling, supporting the hypothesis of CD82 as a microenvironment-dependent immunoregulatory molecule. More importantly, a quadruple-positive T cell subset (CD8^+^CD82^+^TIM-3^+^PD-1^+^) is identified as an independent prognostic risk factor. A high proportion of this subset is significantly associated with reduced patient survival, providing a potential means to optimize existing prognostic models for colon cancer (32, 33).

Regarding immune therapy response, the high expression of CD82 in T cells from non-pCR patients and its association with exhausted subsets suggest that CD82 may serve as a potential biomarker for predicting resistance to immunotherapy (34). At the mechanistic level, the transcription factors BATF and BHLHE40 likely drive CD82 expression by directly binding to its regulatory regions. This is consistent with established knowledge: BATF contributes to T cell exhaustion by upregulating exhaustion-associated genes, while BHLHE40 may promote the terminal differentiation of exhausted T cells (5, 35, 36). Therefore, targeting CD82 or its upstream regulators (e.g., BATF/BHLHE40) could help reverse T cell exhaustion and enhance the efficacy of existing immune checkpoint inhibitors. This study is the first to integrate CD82 into the regulatory network governing immunotherapy response in colon cancer, offering novel targets and insights for overcoming resistance.

This study has several limitations that warrant further investigation. First, the retrospective design limits the ability to infer causality directly. Although statistically significant associations are observed, the specific biological function of CD82 in T cell exhaustion requires validation through prospective cohort studies and functional experiments. In particular, direct functional assays such as CD82 knockdown or overexpression in T cells are not included in the current study, and this represents an important limitation that prevents definitive mechanistic confirmation. Technologically, while mIHC reveals cellular spatial distribution, the interaction network between CD82^+^ cells and tumor or other immune cells requires in-depth analysis using high-dimensional imaging techniques (e.g., CODEX or IMC). At the mechanistic level, the hypothesis that BATF/BHLHE40 transcriptionally regulates CD82 requires validation through ChIP-qPCR, reporter gene assays, and CRISPR-mediated gene editing in both *in vitro* and *in vivo* models. Furthermore, as the conclusions of this study are based on a colon cancer cohort, their applicability to other gastrointestinal tract cancers (e.g., gastric or pancreatic cancer) requires cross-cancer type validation.

To address these limitations, future research may proceed as follows: First, a conditional CD82 knockout model in T cells should be established and combined with adoptive T cell transfer experiments to clarify the functional role of CD82 in T cell exhaustion. Complementary gain- and loss-of-function assays, including CD82 overexpression and knockdown, will also be essential to verify the direct regulatory effects of CD82 on T cell phenotype and function. Second, CD82-targeting blocking antibodies or small-molecule compounds should be developed, and their synergistic effects with anti-PD-1/PD-L1 therapy should be evaluated. Third, multicenter clinical validation of the CD8^+^CD82^+^TIM-3^+^PD-1^+^ quadruple-positive T cell subset as a prognostic biomarker should be advanced to explore its potential for translational application.

In summary, this study establishes CD82 as a key molecule associated with T cell exhaustion in colon cancer, with its co-expression pattern with key immune checkpoints demonstrating significant prognostic value. The CD8^+^CD82^+^TIM-3^+^PD-1^+^ T cell subset serves as an independent risk factor to optimize existing prognostic assessment systems, while targeting the BATF/BHLHE40–CD82 signaling axis could provide new strategies for reversing immunotherapy resistance. These findings deepen the understanding of dynamic regulatory mechanisms within the tumor immune microenvironment and provide new theoretical foundations and intervention strategies for precision immunotherapy in colon cancer.

## Data Availability

The datasets presented in this study can be found in online repositories. The names of the repository/repositories and accession number(s) can be found in the article/supplementary material.
